# Nicotinamide promotes pancreatic differentiation through the dual inhibition of CK1 and ROCK kinases in human embryonic stem cells

**DOI:** 10.1186/s13287-021-02426-2

**Published:** 2021-06-25

**Authors:** Yumeng Zhang, Jiaqi Xu, Zhili Ren, Ya Meng, Weiwei Liu, Ligong Lu, Zhou Zhou, Guokai Chen

**Affiliations:** 1 Centre of Reproduction, Development and Aging, Faculty of Health Sciences, University of Macau, Macau SAR, China; 2 Institute of Translational Medicine, Faculty of Health Sciences, University of Macau, Macau SAR, China; 3 Zhuhai Precision Medical Center, Zhuhai People’s Hospital, Jinan University, Zhuhai, Guangdong China; 4 Bioimaging and Stem Cell Core Facility, Faculty of Health Sciences, University of Macau, Macau SAR, China; 5 State Key Laboratory of Cardiovascular Disease, Beijing Key Laboratory for Molecular Diagnostics of Cardiovascular Diseases, Diagnostic Laboratory Service, Fuwai Hospital, National Center for Cardiovascular Diseases, Chinese Academy of Medical Sciences and Peking Union Medical College, Beijing, China; 6 MoE Frontiers Science Center for Precision Oncology, University of Macau, Macau SAR, China

**Keywords:** Human embryonic stem cells, Pancreatic progenitors, Nicotinamide, Casein kinase 1 (CK1), Rho-associated protein kinase (ROCK), Kinase inhibitor

## Abstract

**Background:**

Vitamin B3 (nicotinamide) plays important roles in metabolism as well as in SIRT and PARP pathways. It is also recently reported as a novel kinase inhibitor with multiple targets. Nicotinamide promotes pancreatic cell differentiation from human embryonic stem cells (hESCs). However, its molecular mechanism is still unclear. In order to understand the molecular mechanism involved in pancreatic cell fate determination, we analyzed the downstream pathways of nicotinamide in the derivation of NKX6.1^+^ pancreatic progenitors from hESCs.

**Methods:**

We applied downstream modulators of nicotinamide during the induction from posterior foregut to pancreatic progenitors, including niacin, PARP inhibitor, SIRT inhibitor, CK1 inhibitor and ROCK inhibitor. The impact of those treatments was evaluated by quantitative real-time PCR, flow cytometry and immunostaining of pancreatic markers. Furthermore, CK1 isoforms were knocked down to validate CK1 function in the induction of pancreatic progenitors. Finally, RNA-seq was used to demonstrate pancreatic induction on the transcriptomic level.

**Results:**

First, we demonstrated that nicotinamide promoted pancreatic progenitor differentiation in chemically defined conditions, but it did not act through either niacin-associated metabolism or the inhibition of PARP and SIRT pathways. In contrast, nicotinamide modulated differentiation through CK1 and ROCK inhibition. We demonstrated that CK1 inhibitors promoted the generation of PDX1/NKX6.1 double-positive pancreatic progenitor cells. shRNA knockdown revealed that the inhibition of CK1α and CK1ε promoted pancreatic progenitor differentiation. We then showed that nicotinamide also improved pancreatic progenitor differentiation through ROCK inhibition. Finally, RNA-seq data showed that CK1 and ROCK inhibition led to pancreatic gene expression, similar to nicotinamide treatment.

**Conclusions:**

In this report, we revealed that nicotinamide promotes generation of pancreatic progenitors from hESCs through CK1 and ROCK inhibition. Furthermore, we discovered the novel role of CK1 in pancreatic cell fate determination.

**Supplementary Information:**

The online version contains supplementary material available at 10.1186/s13287-021-02426-2.

## Background

Unexpected side effects of a specific drug often imply additional targets. These off-target phenomena are valuable resources to reveal novel molecular mechanisms and could help to identify new applications of clinically approved drugs. Human embryonic stem cells (hESCs) are pluripotent and can be differentiated into all cell types. hESC differentiation provides an excellent platform for people to examine key biological processes [1–4]. By delineating the molecular mechanisms of off-target effects in stem cell differentiation, new stem cell applications could be developed from known modulators.

Nicotinamide (NAM) is a multi-target drug that is widely used as a topical treatment for acne, eczema and other skin conditions. Nicotinamide belongs to the vitamin B_3_ family that also includes niacin. Vitamin B_3_ are converted to the coenzyme nicotinamide adenine dinucleotide (NAD) that is essential for energy metabolism [5]. Micromolar-range nicotinamide is sufficient to carry out metabolic functions. When its concentration is elevated to millimolar level, nicotinamide becomes a multi-target compound that is widely utilized in disease treatments and cell culture [6]. Nicotinamide can regulate DNA repair and apoptosis by inhibiting NAD^+^-dependent enzymes such as poly (ADP-ribose) polymerases (PARP) [7]. Nicotinamide can also inhibit Sirtuins (SIRT) to influence epigenetic modification and metabolism [8]. Because of the complexity of kinase cascades and their crosstalk with other pathways, much is unknown about nicotinamide’s function as a kinase inhibitor in disease treatments and cell culture.

Nicotinamide has been widely applied in various aspects of stem cell culture for hESC and human-induced pluripotent stem cells (hiPSC). Nicotinamide suppresses apoptosis and improves reprogramming efficiency [9, 10]. Nicotinamide is also beneficial for the differentiation toward different somatic cell types, such as CD34^+^ hematopoietic progenitors [11], retinal pigment epithelium (RPE) [12], and cardiomyocytes from hESC or hiPSC [13]. hESCs have been induced to pancreatic progenitors, putative β cells, or bona fide β cells by nicotinamide [14–19]. Nicotinamide promotes the development and self-renewal of murine pancreatic progenitors [20], and it also sustains the expression of pan-pancreas marker PDX1 [21] and endocrine marker NKX6.1 in the development of human pancreatic progenitors in serum-containing conditions [22]. However, the mechanism of nicotinamide in pancreatic lineage differentiation remains unclear. Although PARP and SIRT pathways are involved in nicotinamide-associated differentiation, little data is available to validate the mechanism of nicotinamide in cell fate determination processes. We recently show that nicotinamide promotes cell survival as a ROCK inhibitor and induces RPE through CK1 inhibition [6].

Nicotinamide is beneficial for pancreatic progenitor induction, but its downstream effector is unclear in this process. In order to understand the mechanism of nicotinamide in pancreatic in vitro development, we established a serum-free, chemically defined platform to analyze its function in pancreatic differentiation. We hope to reveal critical signaling pathways in pancreatic progenitor differentiation and develop novel methods to generate pancreatic cells from human pluripotent stem cells.

## Materials and methods

### hESC culture

Human ESCs (H1 and H9 lines from WiCell Research Institute, Inc., Madison, WI, http://www.wicell.org) were cultured in Matrigel-coated 6-well plates in E8 medium [23, 24]. Medium was changed daily, and cells were passaged every 3–4 days before they reach 60–70% confluence.

### Pancreatic progenitor differentiation

**Fig. 1:**
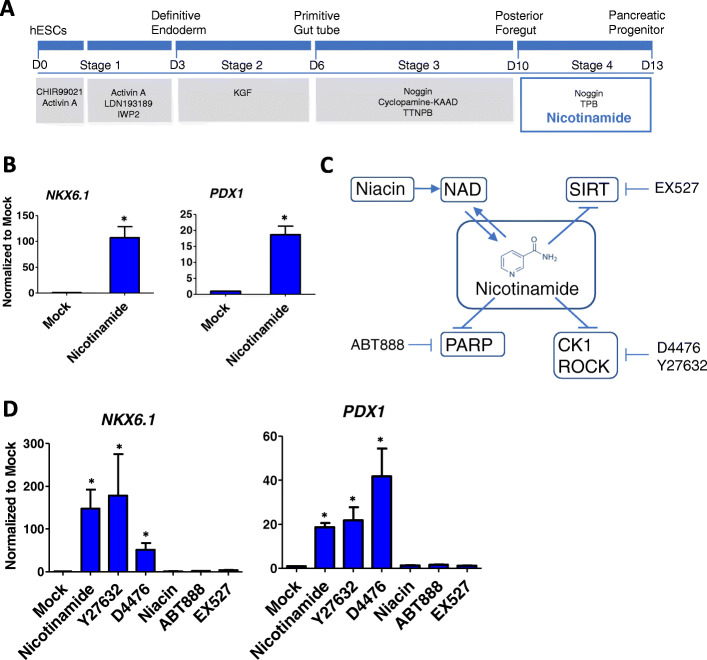
Enhancement of pancreatic progenitor induction by nicotinamide depends on kinase inhibition, but not on PARP and SIRT regulation. **a** Four-stage differentiation strategy to induce pancreatic progenitors from hESCs; **b** RT-qPCR analysis for mRNA levels of *NKX6.1* and *PDX1* in cells on day 13 of differentiation, treated with nicotinamide (n = 3), *p < 0.05; **c** Nicotinamide inhibits PARP, SIRT and several kinases, and contributes to NAD synthesis; **d** RT-qPCR analysis for mRNA level of *NKX6.1* and *PDX1* in cells on day 13 treated with nicotinamide (10 mM), Y27632 (10 μM), D4476 (5 μM), niacin (5 mM), PARP inhibitor ABT888 (50 nM) and SIRT inhibitor EX527 (10 μM) (n = 3), *p < 0.05

The differentiation was carried out in serum-free conditions as illustrated in Fig. 1a. Sixty to 70% confluent hESCs were dissociated with EDTA, passaged onto Matrigel-coated 24-well plate (passaging ratio 1:24), and cultured until 40–50% confluence. Cells were then treated with 5 μM CHIR99021 and 100 ng/ml Activin A in differentiation medium (DMEM/F12, transferrin, chemically defined lipid concentrate, ascorbic acid, and sodium selenite) for 24 h, followed by 100 ng/ml Activin A, 100 nM LDN193189, and 2 μM IWP2 treatment in differentiation medium for another 48 h to induce definitive endoderm formation (Stage 1). Subsequently, primitive gut tube cells were induced under 50 ng/ml KGF in DMEM/F12 with 0.2% NaHCO_3_ and 1× GlutaMAX (Stage 2). Posterior foregut cells were induced under 100 ng/ml Noggin, 1 mM cyclopamine-KAAD, 10 μM TTNPB treatment in DMEM HG with 1% B27 (without insulin), and 1× GlutaMAX (Stage 3). Subsequently, pancreatic progenitors were generated under 100 ng/ml Noggin and 500 nM TPB treatment in DMEM HG with 1% B27 (without insulin), 1× GlutaMAX, and factors involved in nicotinamide-associated pathways as specified (Stage 4). Chemicals used include nicotinamide (10 mM), niacin (1, 5, 10 mM), PARP inhibitor ABT888 (10, 50, 500 nM), SIRT inhibitor EX527 (1, 10, 20 μM), ROCK inhibitor Y27632 (10 μM), CK1 inhibitor D4476 (5 μM), PF4800567 (10 μM) and LH846 (10 μM).

### Real-time PCR

Total mRNA was extracted with RNAiso-plus (TAKARA, cat. no.108-95-2), and reverse transcription from mRNA to cDNA was performed with High Capacity cDNA Reverse Transcription kit (Applied Biosystems, cat. no. 4368813) following the manufacturer’s instructions. Real-time quantitative PCR was conducted with SYBR Premix Ex Taq (TAKARA, cat. no. RR420) and the Quantstudio-7 system (Applied Biosystems). The relative amounts of the amplified nucleotide fragment were calculated by the 2^(-ΔCt) method. Expression levels were normalized to the housekeeping gene TBP and compared with undifferentiated hESCs.

### Immunostaining

Putative pancreatic progenitors were fixed with 4% paraformaldehyde at room temperature for 20 min, rinsed with 1× PBS 3 times, permeabilized with 0.5% Triton X-100 for 20 min, and then stained with primary and secondary antibodies following standard protocols. Primary antibodies include goat anti-PDX1 (Cell Signaling, cat. no. 5679 at 1:2000 dilution) and mouse anti-NKX6.1 (BD biosciences, cat. no. 563338 at 1:200 dilution). The nuclei were stained with Hoechst 33342 (Abnova, cat. no. U0334 at 1:10,000 dilution). Stained cells were visualized using Zeiss Axio Observer fluorescence microscope with ApoTome.

### Flow cytometry

Putative pancreatic progenitors were harvested by TrypLE (37 °C, 5 min) and neutralized with 5% FBS. After washing with DPBS, cells were permeabilized with 0.2% Triton X-100, 5% FBS in DPBS for 1 h on ice. Primary antibodies goat anti-human PDX1 (Cell Signaling, cat. no. 5679 at 1:1000 dilution) and mouse anti-human NKX6.1 conjugated by Alexa Fluor® 647 (BD Biosciences, cat. no. 563338 at 1:20 dilution) were incubated with cells in 1% BSA in DPBS for 1 h at room temperature in the dark. After washing, Alexa Fluor® 488-conjugated anti-goat secondary antibody was used at 1:1000 dilution in 1% BSA in DPBS for 1 h at room temperature in the dark for PDX1 staining. After washing, cells were resuspended in DPBS for analysis using BD ACCURI C6. Undifferentiated hESCs were stained with PDX1 or NKX6.1 as a negative control for gating.

### Western blot

Cells were lysed with RIPA buffer supplemented with phosphatase inhibitor cocktail (Sigma-Aldrich, cat. No. P5726) and proteinase inhibitors. The lysate was sonicated and heated at 95 °C before loading onto 12% sodium dodecyl sulfate-polyacrylamide gel (SDS-PAGE), and the separated proteins were transferred to PVDF membrane. The membrane was blocked with 5% BSA in TBST at room temperature for 30 min, washed with TBST (5 min each time, 3 times), incubated with primary antibodies at 4 °C overnight, washed with TBST (5 min each time, 3 times), and incubated with peroxidase-conjugated secondary antibodies. Signal strength was semi-quantitatively determined by optical densitometry using ImageJ Lab. Primary antibodies include phospho-β-catenin (Ser45) (Cell Signaling, cat. no. 9561 s at 1:1000 dilution), β-catenin (Upstate, cat. no. 06-734 at 1:1000 dilution), phospho-MLC (Ser19) (Cell Signaling, cat. no. 3675 s at 1:2000 dilution), MLC (Sigma-Aldrich, cat. no. SAB1403431 at 1:2000 dilution) and GAPDH (Santa Cruz, cat. no. sc-25778 at 1:2000 dilution).

### Cloning and generation of shRNA in hESCs lines

The psi-LVRU6P plasmid was utilized to create shRNA-expressing constructs. Target sites of shCK1α, shCK1δ and shCK1ɛ are GGAAGTGGCAGTGAAGCTAGA, GGTCCTTCGGAGATATCTACC and GGTTGCCATCAAGCTTGAATG. The lentivirus particles were produced in 293FT cells transfected with psPAX2, pMD2.G and psi-LVRU6P-shRNA-containing plasmids. H1 cells were transduced with the lentivirus and stable cell lines were established by puromycin selection.

### RNA sequencing

Total mRNA was extracted on day 13 (stage 4) using RNAiso-plus (TAKARA, cat. no.108-95-2). The RNA libraries were generated using TruSeq RNA Sample Preparation kit (Illumina), and cDNA fragments were enriched by PCR using Illumina TruSeq PCR primers. Each library was sequenced as paired-end reads in HiSeq 2000/1000 (Illumina). The sequencing data of this report have been uploaded in NCBI’s BioProject and Sequence Read Archive (SRA), and these data are accessible through BioProject accession number PRJNA701836 or SRA accession number SRP306377. 

### Bioinformatics analysis

Transcripts Per Million (TPM) were used to normalize gene read counts in all samples. Values for the heat map were calculated by log2 (TPM of each gene in each sample/mean TPM of each gene in all samples). R package gplots was used to generate heatmaps. R package EdgeR was used to pick differentially expressed genes (DEG) with p value < 0.01, log2 (fold change) > 2 or < − 2. The Euclidean distances between selected genes in each sample were used as the clustering method for the heatmap cluster. Cell type enrichment analysis was performed using the section of “GTEx Tissue Sample Gene Expression Profiles up” in Enrichr (https://maayanlab.cloud/Enrichr/).

### Statistical analysis

Data represent mean ± SEM of ≥ 3 independent experiments unless specified. Statistical significance was tested by either paired t test or one-way ANOVA analysis. *p* < 0.05 represents statistical significance.

## Result

### Enhancement of pancreatic progenitor induction by nicotinamide depends on kinase inhibition, but not on PARP and SIRT regulation

Nicotinamide has been used to induce pancreatic progenitors from hESCs [21, 22], but its molecular mechanism is unclear. In order to examine the function of nicotinamide, a serum-free platform was established to stepwisely induce pancreatic progenitors (Fig. 1a). After H1 hESCs differentiated to posterior foregut (Stage 3), noggin and TPB were applied to induce pancreatic progenitors (Stage 4). We showed that the addition of nicotinamide in stage 4 significantly enhanced the expression of pancreatic progenitor markers *NKX6.1* and *PDX1* (Fig. 1b). This system was utilized to study nicotinamide regulation of pancreatic specification in this report.

Nicotinamide is best known to serve as the substrate to produce NAD as well as an inhibitor for PARP and SIRT pathways (Fig. 1c). Niacin was tested in Stage 4, but it failed to enhance pancreatic differentiation as shown by the mRNA levels of *PDX1* and *NKX6.1* and percentage of PDX1^+^/NKX6.1^+^ cells (Fig. 1d, Fig. S1c, d). It suggested that nicotinamide did not modulate differentiation through NAD metabolism. When PARP inhibitor (ABT888) and SIRT inhibitor (EX527) were applied during Stage 4, they did not improve *NKX6.*1 and *PDX1* mRNA levels and percentage of PDX1^+^/NKX6.1^+^ cells (Fig. 1d, Fig. S1c, d). It indicated that nicotinamide did not modulate pancreatic differentiation through PARP and SIRT inhibition.

We recently found that nicotinamide was also a kinase inhibitor with multiple targets including ROCK and CK1 (Fig. S1a) [6], so we examined whether nicotinamide modulates pancreatic differentiation as a kinase inhibitor. When CK1 inhibitor D4476 and ROCK inhibitor Y27632 were individually applied in stage 4, each of them significantly increased the expression of *PDX1* and *NKX6.1*, and this phenotype was similar to that of nicotinamide (Fig. 1d, Fig. S1e). Besides the H1 cell line, we showed that H9 hESCs can also be induced to pancreatic progenitors as shown by the significant increase of the mRNA levels of *PDX1* and *NKX6.1* upon CK1 or ROCK inhibition (Fig. S1b). These results indicated that nicotinamide influenced pancreatic differentiation as a kinase inhibitor. The cell fate determination could be carried out through CK1 and ROCK in parallel.

### Generic chemical inhibition of CK1 promotes pancreatic progenitor induction

**Fig. 2:**
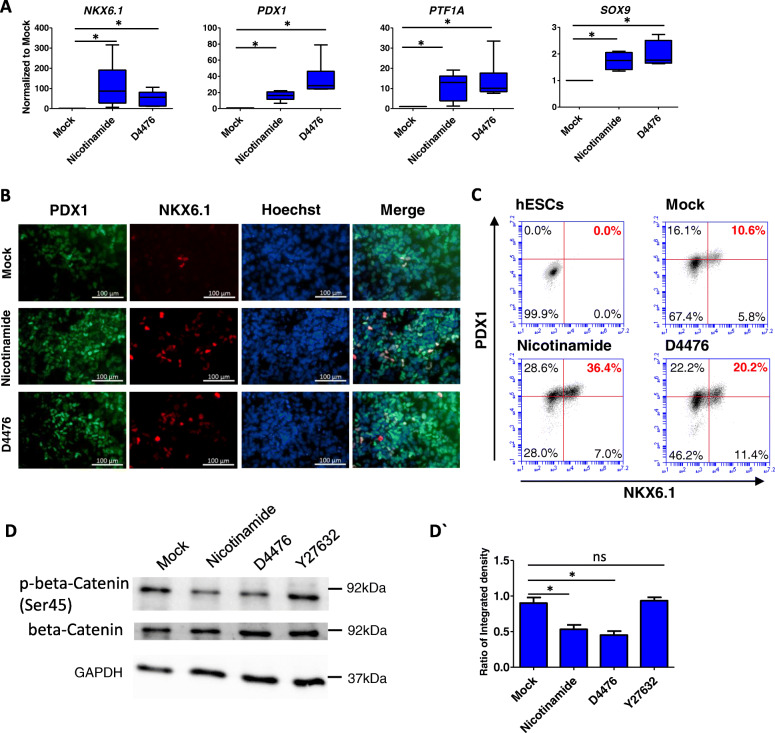
Generic chemical inhibition of CK1 promotes pancreatic progenitor induction. **a** RT-qPCR analysis of pancreatic progenitors on day 13 treated with nicotinamide and D4476 to measure mRNA levels of *NKX6.1*, *PDX1*, *PTF1A* and *SOX9* (n > 3), *p < 0.05; **b** Immunostaining for PDX and NKX6.1 expression in pancreatic progenitors on day 13 under Mock, nicotinamide and D4476 conditions, scale bar 100 μm (n = 3); **c** Flow cytometry analysis of pancreatic progenitors on day 13 for PDX1^+^ and NKX6.1^+^ percentage under Mock, nicotinamide and D4476 conditions (n = 3); **d**, **d`** Western blot analysis of p-β-Catenin (Ser45) in pancreatic progenitors on day 13 under Mock, nicotinamide, D4476 and Y27632 conditions (n = 3), *p < 0.05

We further examined how nicotinamide and CK1 affected pancreatic progenitor differentiation in Stage 4 (Fig. 1a). Similar to nicotinamide, CK1 inhibitor D4476 not only significantly increased the expression of *NKX6.1* and *PDX1*, but also promoted the expression of additional pancreatic progenitor marker genes *PTF1A* and *SOX9* as well as endocrine precursor marker *NGN3* (Fig. 2a, Fig. S2a). Immunostaining results demonstrated that nicotinamide and D4476 both increased the expression of PDX1 and NKX6.1 (Fig. 2b), which were consistent with flow cytometry results (Fig. 2c). These results supported our hypothesis that nicotinamide induced pancreatic progenitors as a CK1 inhibitor.

We then inspected whether downstream factors of CK1 were responsible for the cell fate induction. Previous study revealed that GSK-3 dependent phosphorylation of β-catenin relies on prior priming phosphorylation of β-catenin at Ser45 by CK1 in canonical Wnt pathway [25]. We showed that both nicotinamide and D4476 suppressed the phosphorylation of β-Catenin (Ser45) in putative pancreatic progenitors (Fig. 2d, d`). This result indicated that nicotinamide and D4476 might promote pancreatic progenitor induction through Wnt pathway.

### Inhibition of CK1α and CK1ε promotes pancreatic progenitor induction

**Fig. 3:**
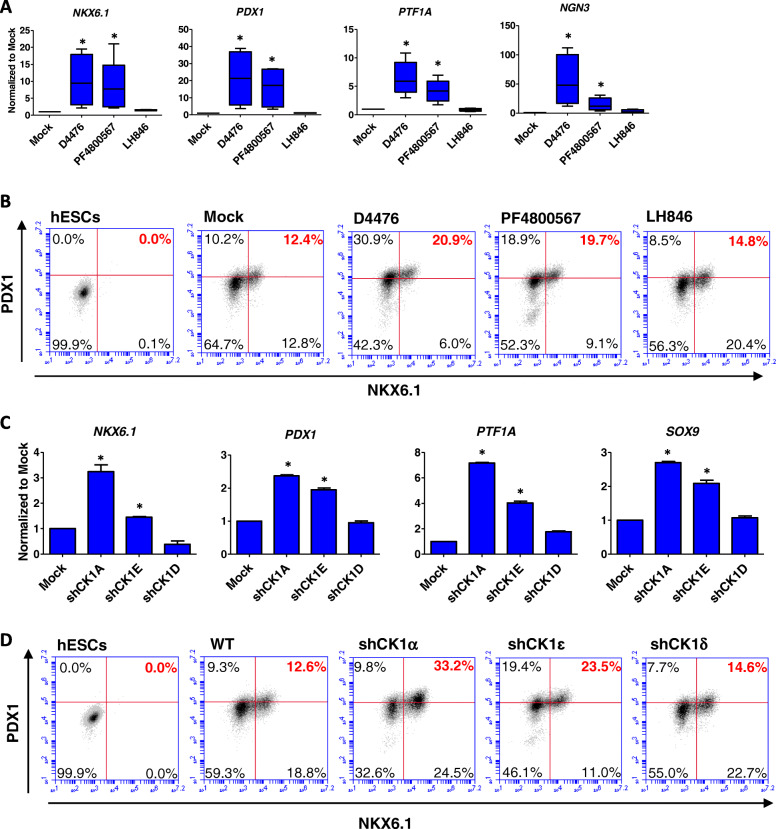
Inhibition of CK1α and CK1ε promotes pancreatic progenitor induction. **a** RT-qPCR analysis of pancreatic progenitors on day 13 treated with D4476, PF4800567 and LH846 to measure mRNA levels of *NKX6.1*, *PDX1*, *PTF1A* and *NGN3* (n > 3), *p < 0.05; **b** Flow cytometry analysis of pancreatic progenitors on day 13 for PDX1^+^ and NKX6.1^+^ percentage under Mock, D4476, PF4800567 and LH846 conditions (n = 3); **c** RT-qPCR analysis of pancreatic progenitors on day 13 induced from shCK1α, shCK1δ and shCK1ε cell lines to measure mRNA level of *NKX6.1* (n = 3), *p < 0.05; **d** Flow cytometry analysis of pancreatic progenitors on day 13 induced from shCK1α, shCK1δ and shCK1ε cell lines for percentage of PDX1^+^ / NKX6.1^+^ (n = 3)

The human genome contains multiple CK1 isoforms with distinct functions. D4476 is a pan CK1 inhibitor that targets CK1α, CK1δ and CK1ε. We tried to determine which CK1 isoform was involved in pancreatic differentiation. CK1 isoform-specific inhibitors were applied in Stage 4 (Fig. 1a), and we showed that CK1ε inhibitor PF4800567 hydrochloride significantly improved the expression of pancreatic progenitor-specific marker genes as well as endocrine precursor marker *NGN3* (Fig. 3a), and it also increased the percentage of PDX1^+^/NKX6.1^+^ cells (Fig. 3b). In contrast, CK1δ inhibitor LH 846 did not significantly improve pancreatic differentiation.

We then used shRNA to knockdown the gene expression of CK1α, CK1δ and CK1ε, respectively (Fig. S3a, b). The knockdown of CK1α and CK1ε significantly upregulated the mRNA levels of typical pancreatic progenitor markers (*NKX6.1*, *PDX1*, *PTF1A* and *SOX9*) (Fig. 3c), and the percentage of PDX1^+^ / NKX6.1^+^ cells was also increased in these two knockdown cell lines (Fig. 3d, Fig. S3c). In contrast, CK1δ knockdown did not improve pancreatic differentiation (Fig. 3c, d, Fig. S3c). These data indicated that CK1α and CK1ε suppressed pancreatic differentiation, which was enhanced by CK1 inhibition.

### Nicotinamide also promotes pancreatic differentiation through ROCK inhibition

**Fig. 4:**
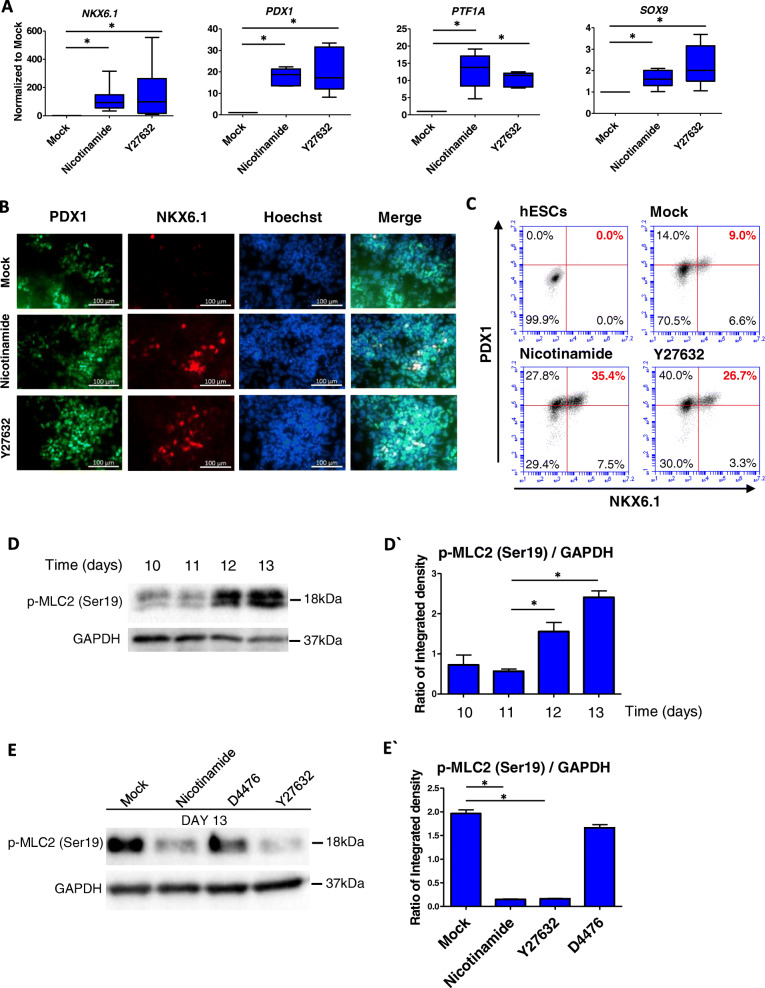
Nicotinamide also promotes pancreatic differentiation through ROCK inhibition. **a** RT-qPCR analysis of pancreatic progenitors on day 13 treated with nicotinamide and Y27632 to measure mRNA levels of *NKX6.1*, *PDX1*, *PTF1A* and *SOX9* (n > 3), *p < 0.05; **b** Immunostaining for PDX and NKX6.1 expression under Mock, nicotinamide and Y27632 conditions, Scale bar 100 μm (n = 3); **c** Flow cytometry analysis of pancreatic progenitors on day 13 for PDX1^+^ and NKX6.1^+^ percentage under Mock, nicotinamide and Y27632 conditions (n = 3); **d, d`** Western blot analysis of p-MLC2 (Ser19) in stage 4 (from day 10 to day 13) (n = 3), *p < 0.05; **e, e`** Western blot analysis of p-MLC2 (Ser19) in day 13 pancreatic progenitors under Mock, nicotinamide, Y27632 and D4476 conditions (n = 3), *p < 0.05

Consistent with previous report on the targets of nicotinamide [6], we found that both CK1 and ROCK were involved in pancreatic progenitor differentiation under nicotinamide treatment (Fig. 1c). We compared the impact of nicotinamide and ROCK inhibitor Y27632 in Stage 4 of pancreatic differentiation. Both nicotinamide and Y27632 significantly improved the expression of pancreatic progenitor genes, including *NKX6.1*, *PDX1*, *PTF1A* and *SOX9* (Fig. 4a), while *NGN3* was only significantly upregulated under nicotinamide treatment. Nicotinamide or Y27632 had no significant impact on the mRNA level of endocrine marker *NEUROD1* (Fig. S4a). Immunostaining showed that the expression of PDX1 and NKX6.1 was upregulated by nicotinamide and Y27632 treatments (Fig. 4b). Flow cytometry results also showed that both nicotinamide and Y27632 upregulated the percentage of PDX1^+^/NKX6.1^+^ double-positive cells (Fig. 4c).

Myosin Light Chain 2 (MLC2) phosphorylation is downstream of ROCK kinase [26], so we evaluated MLC2 phosphorylation at Ser19 during Stage 4 (from day 10 to day 13). Under Noggin/TPB treatment, MLC2 phosphorylation gradually increased from day 10 to day 13 (Fig. 4d, d`). Both nicotinamide and Y27632 significantly decreased MLC2 phosphorylation. However, D4476 did not significantly suppress MLC2 phosphorylation (Fig. 4e, e`). These data suggest that CK1 and ROCK regulate pancreatic differentiation through distinct pathways.

We then modulated pancreatic differentiation through different combinations of ROCK and CK1 inhibition. We showed that the combination of ROCK and CK1 inhibition led to a more significant increase of *PDX1* and *PTF1A* expression, in comparison with ROCK inhibitor alone (Fig. S4b). When Y27632 or D4476 was applied to cells in the presence of nicotinamide, neither of them had an additive effect in the differentiation of PDX1^+^/NKX6.1^+^ cells (Fig. S4c). These results suggest that nicotinamide may induce pancreatic differentiation through a synthetic effect of CK1 and ROCK inhibition.

### Global gene expression of pancreatic progenitors under different treatments

**Fig. 5:**
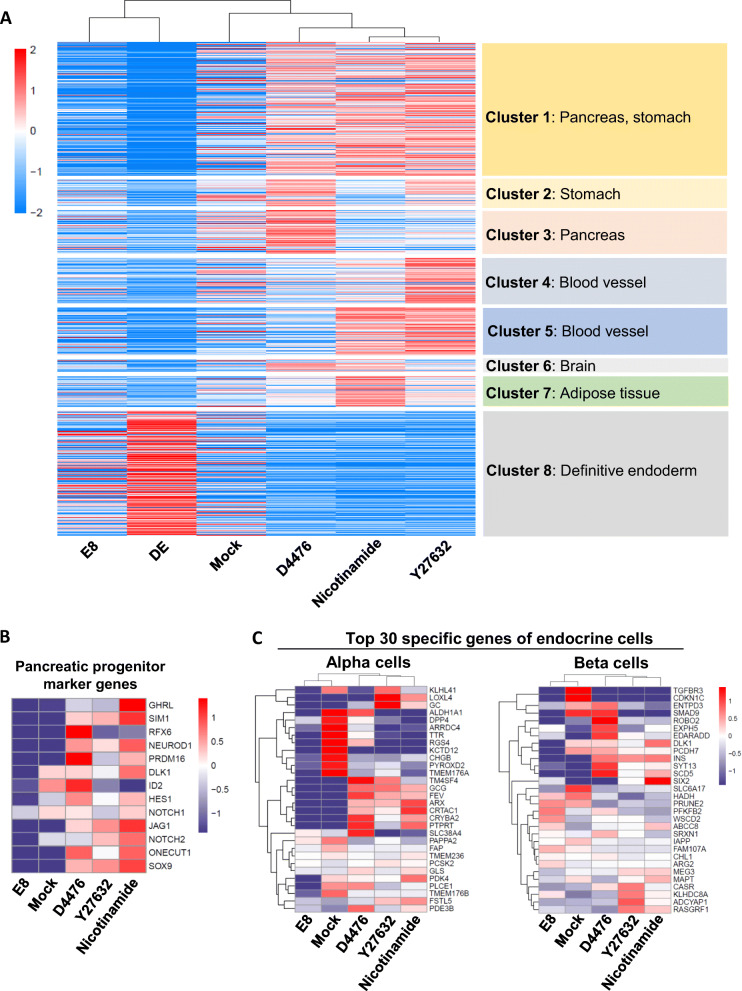
Global gene expression of pancreatic progenitors under different treatments. **a** Heatmap of genes upregulated by D4476, Y27632 and nicotinamide treatments and analysis of cell types by Enrichr. Cluster 1, 2, 3, 4, 5, 6, 7 and 8 contain 1071, 206, 344, 364, 376, 96, 243 and 999 genes, respectively; **b** Heatmap of representative pancreatic progenitor marker genes under Mock, D4476, Y27632, and nicotinamide conditions; **c** Heatmap and hierarchical clustering of representative genes of endocrine alpha, beta cells under Mock, D4476, Y27632 and nicotinamide conditions compared to hESCs cultured in E8

In order to evaluate the pancreatic progenitors induced by different methods, RNA-seq profiles were obtained to examine global gene expression. In comparison to the DE condition, genes in cluster 1 (representing pancreas and stomach) were significantly upregulated by D4476, Y27632 and nicotinamide treatments, and the highly upregulated genes such as *FOXA1*, *JAG1*, *ANKRD1*, *PFKFB3*, *PRSS23*, *CDH6*, *IRS1*, *TANC1*, *LAMC1*, *LAMC2* and *CSRP1* were related to the pancreas. Meanwhile, genes in cluster 2 (representing stomach) were upregulated by D4476 or Y27632 treatment, but not by nicotinamide treatment. Genes in cluster 3 (representing pancreas) were only upregulated by D4476 treatments (Fig. 5a), and the genes of pancreatic organogenesis such as *PRDM16*, *GATA2* and *MAOA* were markedly upregulated. These data indicated that specific CK1 inhibition could be a useful approach to induce a subset of pancreatic marker genes in future applications. We further showed that most pancreatic progenitor markers are upregulated in nicotinamide, CK1 inhibition and ROCK inhibition conditions (Fig. 5b). We analyzed representative gene expression in pancreatic sub-cell types by analyzing published data of a single cell analysis of the human pancreas [27]. The top 30 endocrine-specific genes were generally upregulated in each treatment. However, D4476 led to enhanced expression of more genes representing Beta cells than Y27632 and nicotinamide, indicating that the putative pancreatic progenitors generated via CK1 inhibition may be more likely to go into endocrine cell fates than ROCK inhibition and nicotinamide (Fig. 5c, Fig S5a). To further evaluate the potential of the pancreatic progenitor cells, we further induced hESCs into β-like cells (Fig. S6a), and immunostaining showed that more insulin-producing cells were generated from nicotinamide, D4476 and Y27632 treated pancreatic progenitors (Fig. S6b).

## Discussion

Nicotinamide is involved in diverse biological processes, but its distinct molecular mechanisms in specific applications are not fully defined. Besides its role in metabolism and epigenetic regulation, nicotinamide is emerging as a potent modulator of kinase cascades. By examining nicotinamide function in hESC differentiation, we revealed that nicotinamide inhibits both CK1 and ROCK pathways to promote pancreatic progenitor cell fate.

Casein kinase 1 (CK1) is a family of serine/threonine kinases that are constitutively active in cells. CK1 isoforms are highly involved in circadian rhythms, nucleo-cytoplasmic shuttling of transcription factors, DNA transcription, and DNA repair. CK1 has been implicated in apoptosis and cell proliferation of pancreatic ductal adenocarcinoma cells, but its role in pancreatic differentiation was unknown [28]. In this study, we showed that the suppression of CK1α and CK1ε promoted pancreatic progenitor differentiation, implying that active CK1 pathway is inhibitory to pancreatic differentiation. Nicotinamide and CK1 inhibition probably acted through WNT signaling [29] to modulate pancreatic cell fate [30]. Recent studies showed that pancreatic progenitors can be categorized into three subgroups, including PDX1^+^/NKX6.1^+^, PDX1^+^/NKX6.1^−^, and PDX1^−^/NKX6.1^+^ cells [31–33]. PDX1^+^/NKX6.1^+^ cells are considered as bona fide beta cell precursors, which are significantly induced by CK1 inhibitor and nicotinamide. More work is necessary to explore the differentiation of the other two subtypes in vitro.

This study also showed that nicotinamide induced pancreatic cell fate as a ROCK inhibitor. ROCK pathway is involved in glucose-stimulated insulin secretion [34] and the disassembly of glucotoxicity-induced stress fibers [35]. Recent reports showed that ROCK inhibition promoted the differentiation of PDX1^+^ posterior foregut cells into pancreatic endoderm fate at low cell density [36]. We previously showed that nicotinamide enhances hESC survival by inhibiting MLC2 phosphorylation as a ROCK inhibitor [6]. In pancreatic differentiation, nicotinamide likely promotes pancreatic progenitor fate through a similar mechanism as a ROCK inhibitor.

Although nicotinamide is best known as vitamin B3 for metabolism and inhibitors in SIRT and PARP pathways, we show that nicotinamide modulates pancreatic differentiation through its “off-target” effect as a kinase inhibitor. By exploring nicotinamide’s regulation of kinase targets, we will not only reveal the novel molecular mechanism, but also develop new methods in stem cell applications.

## Conclusion

We demonstrated that the promotion of pancreatic progenitor differentiation by nicotinamide was through the dual inhibition of ROCK and CK1. These findings should be of broad interest to the stem cell community and regenerative medicine. Elucidating the mechanism of pancreatic progenitor and endocrine cell development is the foundation for in vitro induction of pancreatic progenitors and endocrine cell types.

## Supplementary Information

**Additional file 1: Supplemental Figure 1**. Enhancement of pancreatic progenitor induction by nicotinamide depends on kinase inhibition, but not on PARP and SIRT regulation. a Nicotinamide`s molecular targets screened via KINOMEscan™ confirmed that nicotinamide can directly bind and inhibit CK1 and ROCK; b RT-qPCR analysis for mRNA levels of *NKX6.1* and *PDX1* in pancreatic progenitors on day 13 of differentiation from H9, treated with nicotinamide, D4476 and Y27632 (n = 3), *p < 0.05; c RT-qPCR analysis of pancreatic progenitors on day 13 for mRNA levels of *NKX6.1* and *PDX1* under Mock, nicotinamide (10 mM), niacin (1, 5, 10 mM), PARP inhibitor ABT888 (10, 50, 500 nM) and SIRT inhibitor EX527 (1, 10, 20 μM) conditions (n = 3); d Flow cytometry analysis to test the effect of nicotinamide (10 mM), niacin (5 mM), ABT888 (50 nM) and EX527 (10 μM) on generation of PDX1^+^/NKX6.1^+^ cells (n = 3); e Flow cytometry analysis to test the effect of nicotinamide (10 mM), D4476 (5 μM) and Y27632 (10 μM) on the generation of PDX1^+^/NKX6.1^+^ cells (n = 3). **Supplemental Figure 2**.Generic chemical inhibition of CK1 promotes pancreatic progenitor induction. a RT-qPCR analysis of pancreatic progenitors on day 13 treated with nicotinamide and D4476 to measure mRNA levels of *NEUROD1* and *NGN3* (n > 3), *p < 0.05. **Supplemental Figure 3**. Inhibition of CK1α and CK1ε promotes pancreatic progenitor induction. a RT-qPCR analysis to measure mRNA levels of *CK1α*, *CK1ε* and *CK1δ* in shCK1α, shCK1ε and shCK1δ cell lines in pluripotency stage (n = 3), *p < 0.05; b Western blot analysis to assay protein level of CK1α, CK1ε and CK1δ in shCK1α, shCK1ε and shCK1δ cell lines in pluripotency stage; c Flow cytometry analysis of pancreatic progenitors on day 13 induced from shCK1α, shCK1δ and shCK1ε cell lines for percentage of PDX1^+^ / NKX6.1^+^ (n = 3). **Supplemental Figure 4.** Nicotinamide also promotes pancreatic differentiation through ROCK inhibition. a RT-qPCR analysis of pancreatic progenitors on day 13 treated with nicotinamide and Y27632 to measure mRNA levels of *NEUROD1* and *NGN3* (n > 3), *p < 0.05; b RT-qPCR analysis of pancreatic progenitors on day 13 treated with nicotinamide, Y27632, D4476 and Y27632+D4476 to measure mRNA levels of *NKX6.1*, *PDX1* and *PTF1A* (n > 3), *p < 0.05; c Flow cytometry analysis to test the effect of nicotinamide, nicotinamide+D4476 and nicotinamide+Y27632 on the generation of PDX1^+^/NKX6.1^+^ cells (n = 3). **Supplemental Figure 5.** Global gene expression of pancreatic progenitors under different treatments. a Heatmap and hierarchical clustering of representative genes of delta, epsilon, pancreatic polypeptide (PP), Acinar and ductal cells under Mock, D4476, Y27632 and nicotinamide conditions compared to hESCs cultured in E8. **Supplemental Figure 6.** Pancreatic progenitors under Mock, nicotinamide, D4476 and Y27632 treatment can be further induced into insulin positive β-like cells. a Differentiation strategy from pancreatic progenitors to β-like cells; b Immunostaining for insulin expression in β-like cells generated from pancreatic progenitors under Mock, nicotinamide, D4476 and Y27632 condition, Scale bar 400 μM and 100 μm.

**Additional file 2. **Primers used in RT-qPCR analysis. 

**Additional file 3. **Antibodies used in flow cytometry, immunostaining or Western Blot analysis.

**Additional file 4. **Chemicals or recombinant proteins used in hESC differentiation. 

## Data Availability

The data supporting the finding of this article are all online.

## Abbreviations

hESCs: Human embryonic stem cells
ROCK: Rho-associated protein kinase
CK1: Casein kinase
MLC2: Myosin light chain 2
NAM: Nicotinamide
NAD: Nicotinamide adenine dinucleotide
PARP: Poly (ADP-ribose) polymerases
SIRT: Sirtuins
PP: Pancreatic polypeptide cells
