# Supplementary figures and images for: Nicotinamide promotes pancreatic differentiation through the dual inhibition of CK1 and ROCK kinases in human embryonic stem cells
Source: Stem Cell Res Ther. 2021 Jun 25;12:362. doi: 10.1186/s13287-021-02426-2 (PMC8235863; doi:10.1186/s13287-021-02426-2)

Figure S1.

A

| Nicotinamide Targets |         |
|----------------------|---------|
| Gene Symbol          | Kd (μM) |
| CSNK1A1              | 546.580 |
| CSNK1E               | 612.076 |
| CSNK1D               | 352.512 |
| ROCK1                | 161.698 |
| ROCK2                | 212.158 |

B

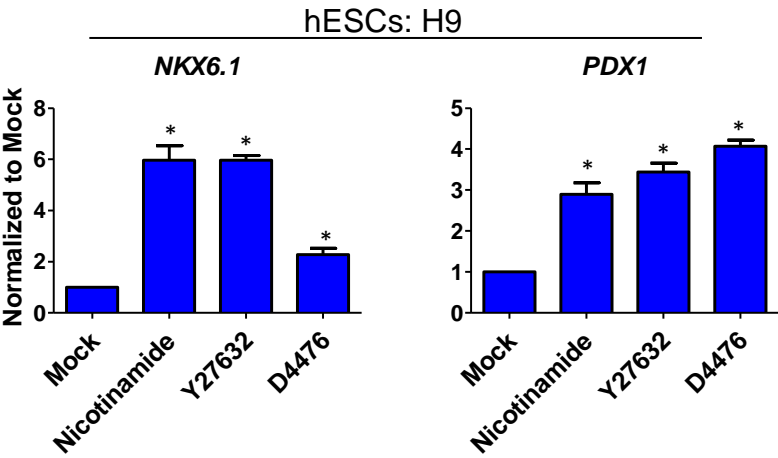

C

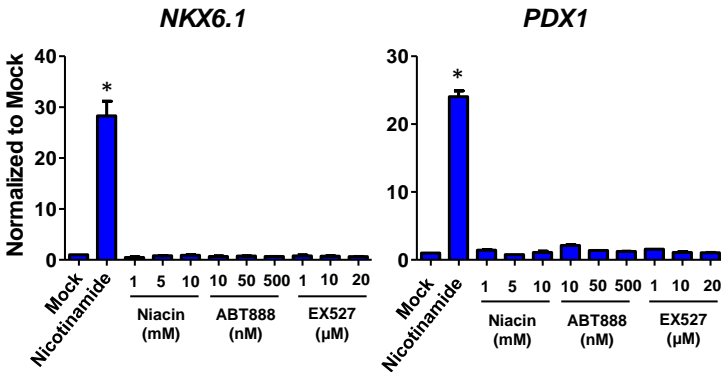

D

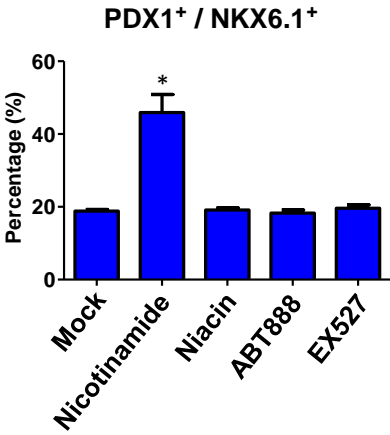

E

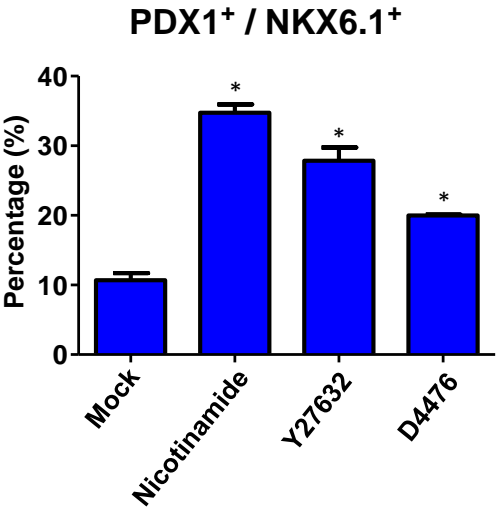

Supplement: Supplementary file 1 — Additional file 1: Supplemental Figure 1. Enhancement of pancreatic progenitor induction by nicotinamide depends on kinase inhibition, but not on PARP and SIRT regulation. a Nicotinamide`s molecular targets screened via KINOMEscan™ confirmed that nicotinamide can directly bind and inhibit CK1 and ROCK; b RT-qPCR analysis for mRNA levels of NKX6.1 and PDX1 in pancreatic progenitors on day 13 of differentiation from H9, treated with nicotinamide, D4476 and Y27632 (n = 3), *p < 0.05; c RT-qPCR analysis of pancreatic progenitors on day 13 for mRNA levels of NKX6.1 and PDX1 under Mock, nicotinamide (10 mM), niacin (1, 5, 10 mM), PARP inhibitor ABT888 (10, 50, 500 nM) and SIRT inhibitor EX527 (1, 10, 20 μM) conditions (n = 3); d Flow cytometry analysis to test the effect of nicotinamide (10 mM), niacin (5 mM), ABT888 (50 nM) and EX527 (10 μM) on generation of PDX1+/NKX6.1+ cells (n = 3); e Flow cytometry analysis to test the effect of nicotinamide (10 mM), D4476 (5 μM) and Y27632 (10 μM) on the generation of PDX1+/NKX6.1+ cells (n = 3). Supplemental Figure 2.Generic chemical inhibition of CK1 promotes pancreatic progenitor induction. a RT-qPCR analysis of pancreatic progenitors on day 13 treated with nicotinamide and D4476 to measure mRNA levels of NEUROD1 and NGN3 (n > 3), *p < 0.05. Supplemental Figure 3. Inhibition of CK1α and CK1ε promotes pancreatic progenitor induction. a RT-qPCR analysis to measure mRNA levels of CK1α, CK1ε and CK1δ in shCK1α, shCK1ε and shCK1δ cell lines in pluripotency stage (n = 3), *p < 0.05; b Western blot analysis to assay protein level of CK1α, CK1ε and CK1δ in shCK1α, shCK1ε and shCK1δ cell lines in pluripotency stage; c Flow cytometry analysis of pancreatic progenitors on day 13 induced from shCK1α, shCK1δ and shCK1ε cell lines for percentage of PDX1+ / NKX6.1+ (n = 3). Supplemental Figure 4. Nicotinamide also promotes pancreatic differentiation through ROCK inhibition. a RT-qPCR analysis of pancreatic progenitors on day 13 treated with ni [file 13287_2021_2426_MOESM1_ESM.zip › 13287_2021_2426_MOESM1_ESM/Supplemental Figure 1_ESM.pdf]

Figure S2.

A

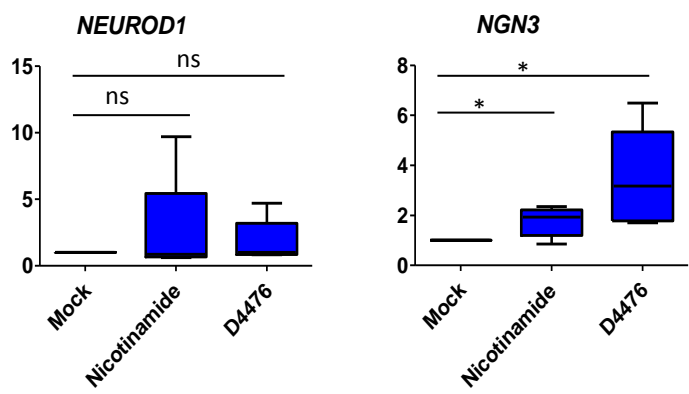

Supplement: Supplementary file 1 — Additional file 1: Supplemental Figure 1. Enhancement of pancreatic progenitor induction by nicotinamide depends on kinase inhibition, but not on PARP and SIRT regulation. a Nicotinamide`s molecular targets screened via KINOMEscan™ confirmed that nicotinamide can directly bind and inhibit CK1 and ROCK; b RT-qPCR analysis for mRNA levels of NKX6.1 and PDX1 in pancreatic progenitors on day 13 of differentiation from H9, treated with nicotinamide, D4476 and Y27632 (n = 3), *p < 0.05; c RT-qPCR analysis of pancreatic progenitors on day 13 for mRNA levels of NKX6.1 and PDX1 under Mock, nicotinamide (10 mM), niacin (1, 5, 10 mM), PARP inhibitor ABT888 (10, 50, 500 nM) and SIRT inhibitor EX527 (1, 10, 20 μM) conditions (n = 3); d Flow cytometry analysis to test the effect of nicotinamide (10 mM), niacin (5 mM), ABT888 (50 nM) and EX527 (10 μM) on generation of PDX1+/NKX6.1+ cells (n = 3); e Flow cytometry analysis to test the effect of nicotinamide (10 mM), D4476 (5 μM) and Y27632 (10 μM) on the generation of PDX1+/NKX6.1+ cells (n = 3). Supplemental Figure 2.Generic chemical inhibition of CK1 promotes pancreatic progenitor induction. a RT-qPCR analysis of pancreatic progenitors on day 13 treated with nicotinamide and D4476 to measure mRNA levels of NEUROD1 and NGN3 (n > 3), *p < 0.05. Supplemental Figure 3. Inhibition of CK1α and CK1ε promotes pancreatic progenitor induction. a RT-qPCR analysis to measure mRNA levels of CK1α, CK1ε and CK1δ in shCK1α, shCK1ε and shCK1δ cell lines in pluripotency stage (n = 3), *p < 0.05; b Western blot analysis to assay protein level of CK1α, CK1ε and CK1δ in shCK1α, shCK1ε and shCK1δ cell lines in pluripotency stage; c Flow cytometry analysis of pancreatic progenitors on day 13 induced from shCK1α, shCK1δ and shCK1ε cell lines for percentage of PDX1+ / NKX6.1+ (n = 3). Supplemental Figure 4. Nicotinamide also promotes pancreatic differentiation through ROCK inhibition. a RT-qPCR analysis of pancreatic progenitors on day 13 treated with ni [file 13287_2021_2426_MOESM1_ESM.zip › 13287_2021_2426_MOESM1_ESM/Supplemental Figure 2_ESM.pdf]

Figure S3.

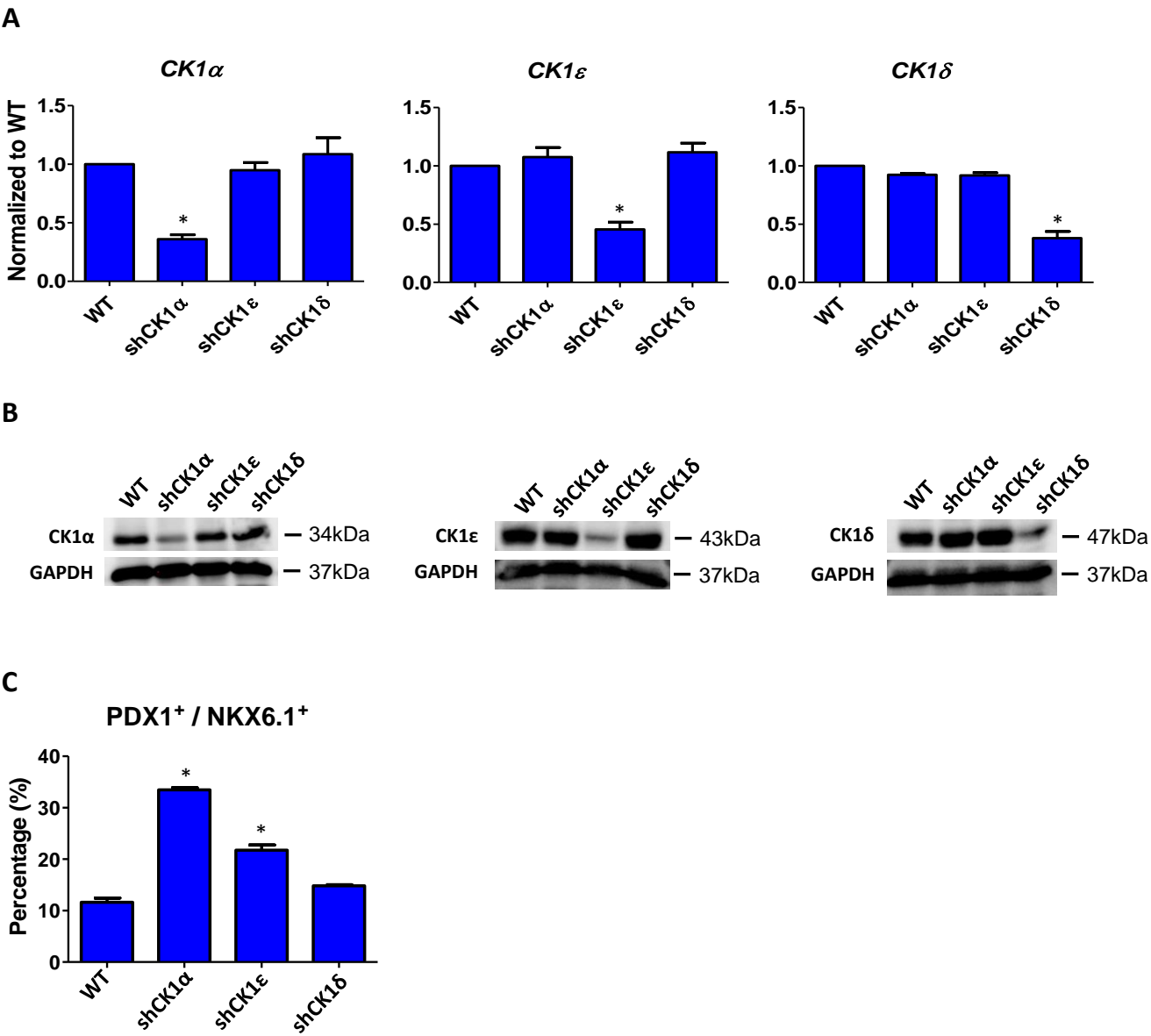

Supplement: Supplementary file 1 — Additional file 1: Supplemental Figure 1. Enhancement of pancreatic progenitor induction by nicotinamide depends on kinase inhibition, but not on PARP and SIRT regulation. a Nicotinamide`s molecular targets screened via KINOMEscan™ confirmed that nicotinamide can directly bind and inhibit CK1 and ROCK; b RT-qPCR analysis for mRNA levels of NKX6.1 and PDX1 in pancreatic progenitors on day 13 of differentiation from H9, treated with nicotinamide, D4476 and Y27632 (n = 3), *p < 0.05; c RT-qPCR analysis of pancreatic progenitors on day 13 for mRNA levels of NKX6.1 and PDX1 under Mock, nicotinamide (10 mM), niacin (1, 5, 10 mM), PARP inhibitor ABT888 (10, 50, 500 nM) and SIRT inhibitor EX527 (1, 10, 20 μM) conditions (n = 3); d Flow cytometry analysis to test the effect of nicotinamide (10 mM), niacin (5 mM), ABT888 (50 nM) and EX527 (10 μM) on generation of PDX1+/NKX6.1+ cells (n = 3); e Flow cytometry analysis to test the effect of nicotinamide (10 mM), D4476 (5 μM) and Y27632 (10 μM) on the generation of PDX1+/NKX6.1+ cells (n = 3). Supplemental Figure 2.Generic chemical inhibition of CK1 promotes pancreatic progenitor induction. a RT-qPCR analysis of pancreatic progenitors on day 13 treated with nicotinamide and D4476 to measure mRNA levels of NEUROD1 and NGN3 (n > 3), *p < 0.05. Supplemental Figure 3. Inhibition of CK1α and CK1ε promotes pancreatic progenitor induction. a RT-qPCR analysis to measure mRNA levels of CK1α, CK1ε and CK1δ in shCK1α, shCK1ε and shCK1δ cell lines in pluripotency stage (n = 3), *p < 0.05; b Western blot analysis to assay protein level of CK1α, CK1ε and CK1δ in shCK1α, shCK1ε and shCK1δ cell lines in pluripotency stage; c Flow cytometry analysis of pancreatic progenitors on day 13 induced from shCK1α, shCK1δ and shCK1ε cell lines for percentage of PDX1+ / NKX6.1+ (n = 3). Supplemental Figure 4. Nicotinamide also promotes pancreatic differentiation through ROCK inhibition. a RT-qPCR analysis of pancreatic progenitors on day 13 treated with ni [file 13287_2021_2426_MOESM1_ESM.zip › 13287_2021_2426_MOESM1_ESM/Supplemental Figure 3_ESM.pdf]

Figure S4.

A

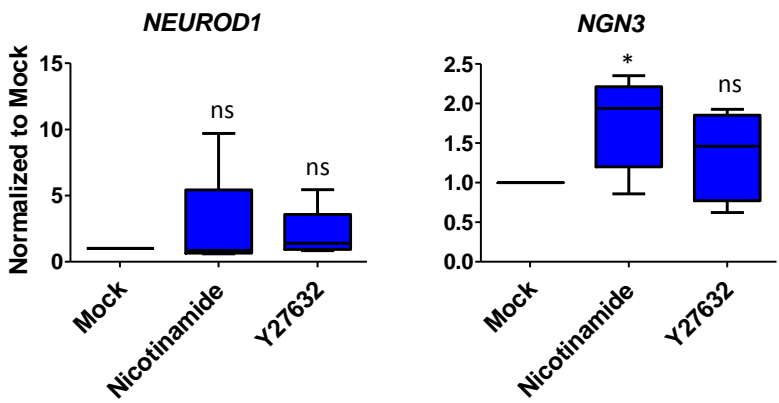

B

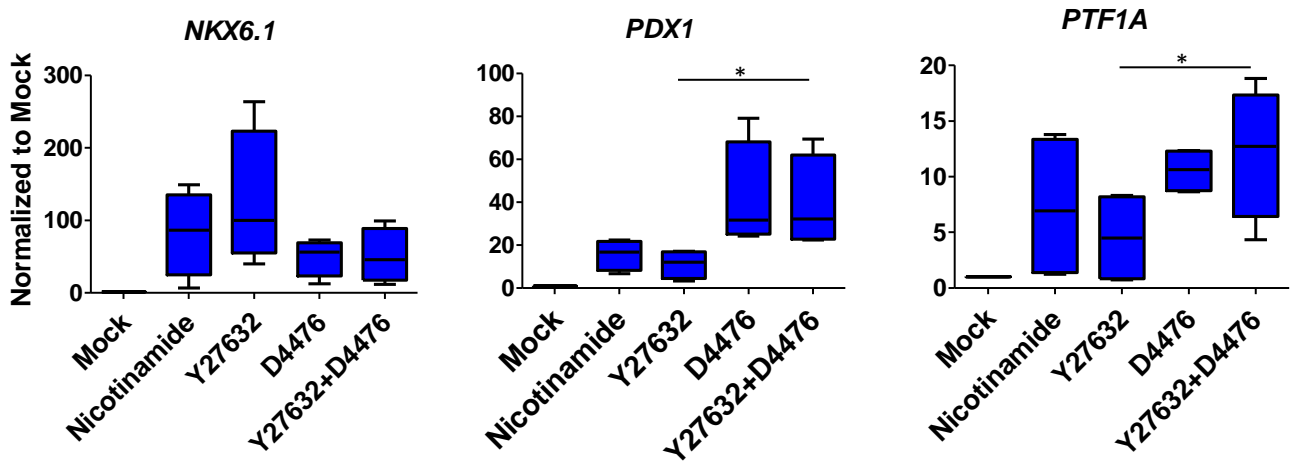

C

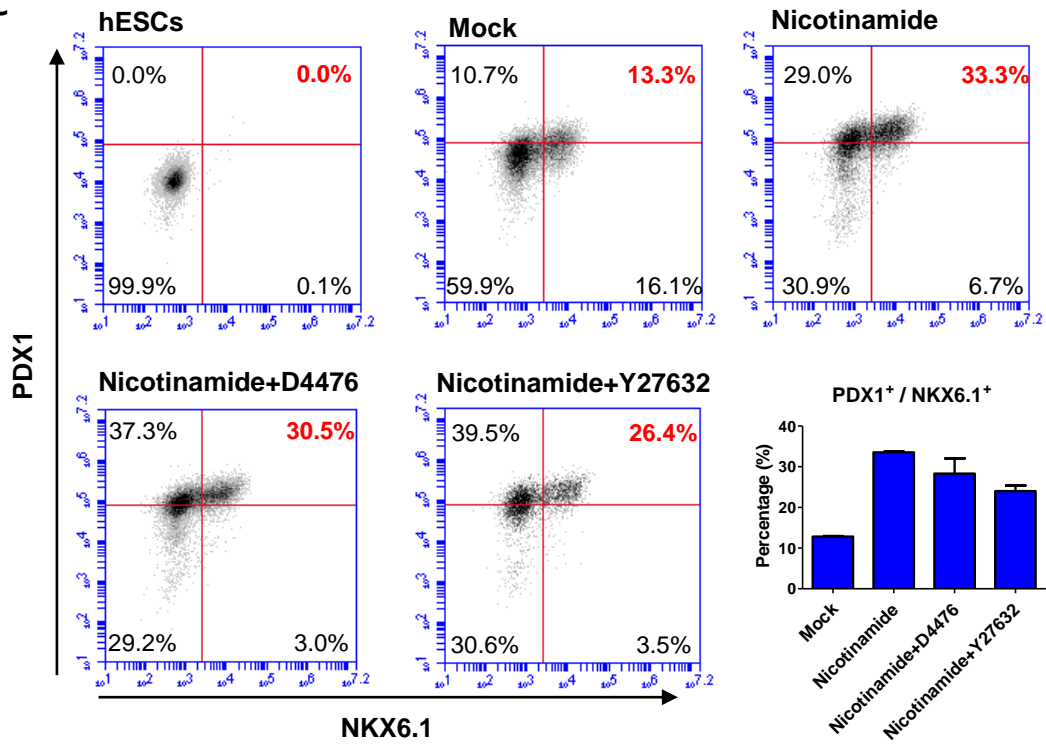

Supplement: Supplementary file 1 — Additional file 1: Supplemental Figure 1. Enhancement of pancreatic progenitor induction by nicotinamide depends on kinase inhibition, but not on PARP and SIRT regulation. a Nicotinamide`s molecular targets screened via KINOMEscan™ confirmed that nicotinamide can directly bind and inhibit CK1 and ROCK; b RT-qPCR analysis for mRNA levels of NKX6.1 and PDX1 in pancreatic progenitors on day 13 of differentiation from H9, treated with nicotinamide, D4476 and Y27632 (n = 3), *p < 0.05; c RT-qPCR analysis of pancreatic progenitors on day 13 for mRNA levels of NKX6.1 and PDX1 under Mock, nicotinamide (10 mM), niacin (1, 5, 10 mM), PARP inhibitor ABT888 (10, 50, 500 nM) and SIRT inhibitor EX527 (1, 10, 20 μM) conditions (n = 3); d Flow cytometry analysis to test the effect of nicotinamide (10 mM), niacin (5 mM), ABT888 (50 nM) and EX527 (10 μM) on generation of PDX1+/NKX6.1+ cells (n = 3); e Flow cytometry analysis to test the effect of nicotinamide (10 mM), D4476 (5 μM) and Y27632 (10 μM) on the generation of PDX1+/NKX6.1+ cells (n = 3). Supplemental Figure 2.Generic chemical inhibition of CK1 promotes pancreatic progenitor induction. a RT-qPCR analysis of pancreatic progenitors on day 13 treated with nicotinamide and D4476 to measure mRNA levels of NEUROD1 and NGN3 (n > 3), *p < 0.05. Supplemental Figure 3. Inhibition of CK1α and CK1ε promotes pancreatic progenitor induction. a RT-qPCR analysis to measure mRNA levels of CK1α, CK1ε and CK1δ in shCK1α, shCK1ε and shCK1δ cell lines in pluripotency stage (n = 3), *p < 0.05; b Western blot analysis to assay protein level of CK1α, CK1ε and CK1δ in shCK1α, shCK1ε and shCK1δ cell lines in pluripotency stage; c Flow cytometry analysis of pancreatic progenitors on day 13 induced from shCK1α, shCK1δ and shCK1ε cell lines for percentage of PDX1+ / NKX6.1+ (n = 3). Supplemental Figure 4. Nicotinamide also promotes pancreatic differentiation through ROCK inhibition. a RT-qPCR analysis of pancreatic progenitors on day 13 treated with ni [file 13287_2021_2426_MOESM1_ESM.zip › 13287_2021_2426_MOESM1_ESM/Supplemental Figure 4_ESM.pdf]

Figure S5.

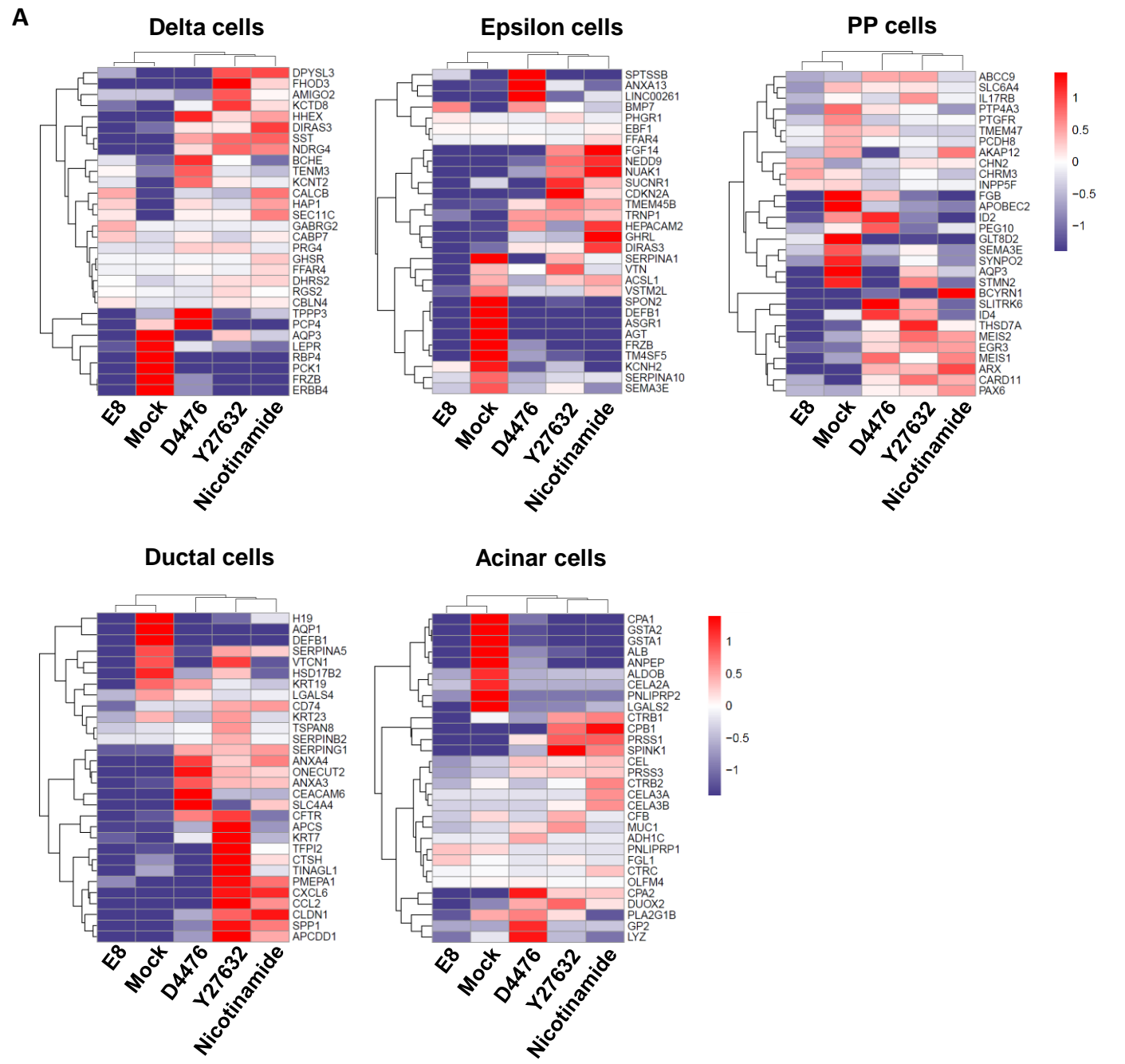

Supplement: Supplementary file 1 — Additional file 1: Supplemental Figure 1. Enhancement of pancreatic progenitor induction by nicotinamide depends on kinase inhibition, but not on PARP and SIRT regulation. a Nicotinamide`s molecular targets screened via KINOMEscan™ confirmed that nicotinamide can directly bind and inhibit CK1 and ROCK; b RT-qPCR analysis for mRNA levels of NKX6.1 and PDX1 in pancreatic progenitors on day 13 of differentiation from H9, treated with nicotinamide, D4476 and Y27632 (n = 3), *p < 0.05; c RT-qPCR analysis of pancreatic progenitors on day 13 for mRNA levels of NKX6.1 and PDX1 under Mock, nicotinamide (10 mM), niacin (1, 5, 10 mM), PARP inhibitor ABT888 (10, 50, 500 nM) and SIRT inhibitor EX527 (1, 10, 20 μM) conditions (n = 3); d Flow cytometry analysis to test the effect of nicotinamide (10 mM), niacin (5 mM), ABT888 (50 nM) and EX527 (10 μM) on generation of PDX1+/NKX6.1+ cells (n = 3); e Flow cytometry analysis to test the effect of nicotinamide (10 mM), D4476 (5 μM) and Y27632 (10 μM) on the generation of PDX1+/NKX6.1+ cells (n = 3). Supplemental Figure 2.Generic chemical inhibition of CK1 promotes pancreatic progenitor induction. a RT-qPCR analysis of pancreatic progenitors on day 13 treated with nicotinamide and D4476 to measure mRNA levels of NEUROD1 and NGN3 (n > 3), *p < 0.05. Supplemental Figure 3. Inhibition of CK1α and CK1ε promotes pancreatic progenitor induction. a RT-qPCR analysis to measure mRNA levels of CK1α, CK1ε and CK1δ in shCK1α, shCK1ε and shCK1δ cell lines in pluripotency stage (n = 3), *p < 0.05; b Western blot analysis to assay protein level of CK1α, CK1ε and CK1δ in shCK1α, shCK1ε and shCK1δ cell lines in pluripotency stage; c Flow cytometry analysis of pancreatic progenitors on day 13 induced from shCK1α, shCK1δ and shCK1ε cell lines for percentage of PDX1+ / NKX6.1+ (n = 3). Supplemental Figure 4. Nicotinamide also promotes pancreatic differentiation through ROCK inhibition. a RT-qPCR analysis of pancreatic progenitors on day 13 treated with ni [file 13287_2021_2426_MOESM1_ESM.zip › 13287_2021_2426_MOESM1_ESM/Supplemental Figure 5_ESM.pdf]

Figure S6.

A

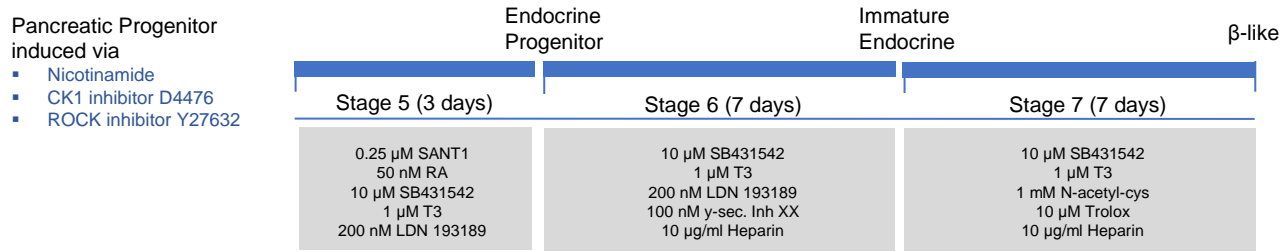

B

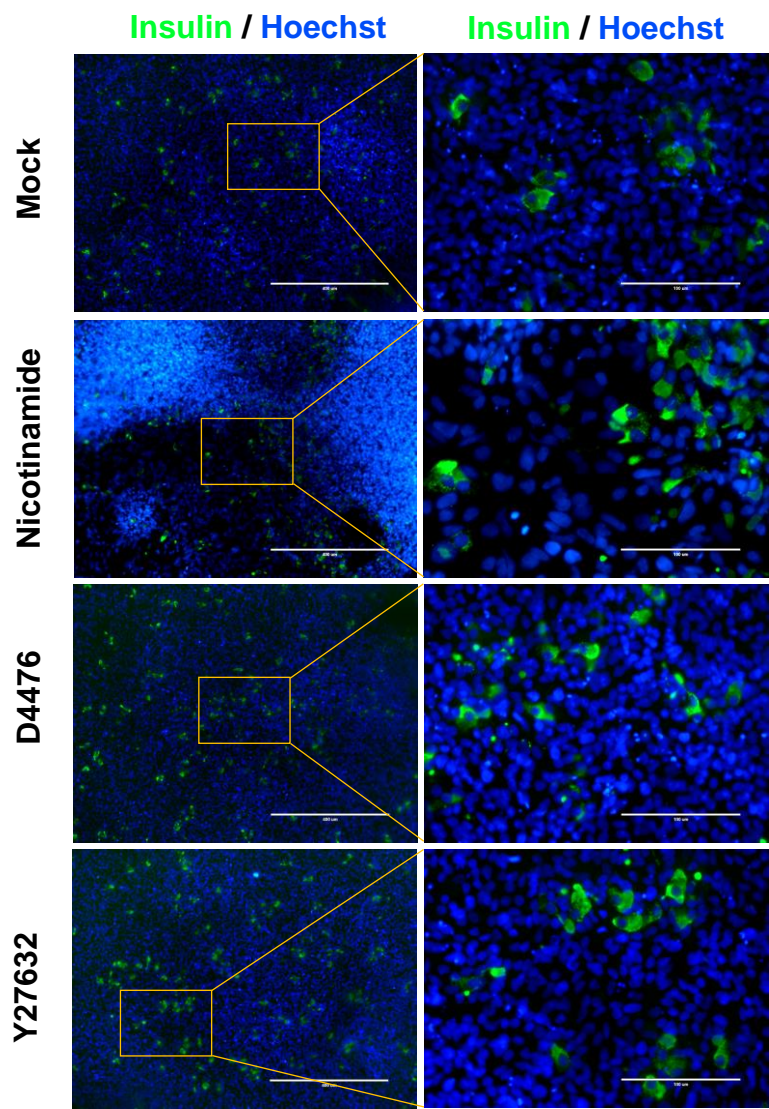

Supplement: Supplementary file 1 — Additional file 1: Supplemental Figure 1. Enhancement of pancreatic progenitor induction by nicotinamide depends on kinase inhibition, but not on PARP and SIRT regulation. a Nicotinamide`s molecular targets screened via KINOMEscan™ confirmed that nicotinamide can directly bind and inhibit CK1 and ROCK; b RT-qPCR analysis for mRNA levels of NKX6.1 and PDX1 in pancreatic progenitors on day 13 of differentiation from H9, treated with nicotinamide, D4476 and Y27632 (n = 3), *p < 0.05; c RT-qPCR analysis of pancreatic progenitors on day 13 for mRNA levels of NKX6.1 and PDX1 under Mock, nicotinamide (10 mM), niacin (1, 5, 10 mM), PARP inhibitor ABT888 (10, 50, 500 nM) and SIRT inhibitor EX527 (1, 10, 20 μM) conditions (n = 3); d Flow cytometry analysis to test the effect of nicotinamide (10 mM), niacin (5 mM), ABT888 (50 nM) and EX527 (10 μM) on generation of PDX1+/NKX6.1+ cells (n = 3); e Flow cytometry analysis to test the effect of nicotinamide (10 mM), D4476 (5 μM) and Y27632 (10 μM) on the generation of PDX1+/NKX6.1+ cells (n = 3). Supplemental Figure 2.Generic chemical inhibition of CK1 promotes pancreatic progenitor induction. a RT-qPCR analysis of pancreatic progenitors on day 13 treated with nicotinamide and D4476 to measure mRNA levels of NEUROD1 and NGN3 (n > 3), *p < 0.05. Supplemental Figure 3. Inhibition of CK1α and CK1ε promotes pancreatic progenitor induction. a RT-qPCR analysis to measure mRNA levels of CK1α, CK1ε and CK1δ in shCK1α, shCK1ε and shCK1δ cell lines in pluripotency stage (n = 3), *p < 0.05; b Western blot analysis to assay protein level of CK1α, CK1ε and CK1δ in shCK1α, shCK1ε and shCK1δ cell lines in pluripotency stage; c Flow cytometry analysis of pancreatic progenitors on day 13 induced from shCK1α, shCK1δ and shCK1ε cell lines for percentage of PDX1+ / NKX6.1+ (n = 3). Supplemental Figure 4. Nicotinamide also promotes pancreatic differentiation through ROCK inhibition. a RT-qPCR analysis of pancreatic progenitors on day 13 treated with ni [file 13287_2021_2426_MOESM1_ESM.zip › 13287_2021_2426_MOESM1_ESM/Supplemental Figure 6_ESM.pdf]
